# Supplementary material for: Traffic signal optimization on a square lattice with quantum annealing
Source: Sci Rep. 2021 Feb 10;11:3303. doi: 10.1038/s41598-021-82740-0 (PMC7875976; doi:10.1038/s41598-021-82740-0)
Supplement: Supplementary file 1 — Supplementary Information. [file 41598_2021_82740_MOESM1_ESM.pdf]

# Supplemental information of "Traffic Signal Optimization on a Square Lattice with Quantum Annealing"

Daisuke Inoue,[1,*] Akihisa Okada,[1] Tadayoshi Matsumori,[1] Kazuyuki Aihara,[2,3] and Hiroaki Yoshida[1]

[1] *Toyota Central R&D Labs., Inc. Bunkyo-ku, Tokyo 112-0004, Japan*
[2] *Institute of Industrial Science, The University of Tokyo, Meguro-ku, Tokyo 153-8505, Japan*
[3] *International Research Center for Neurointelligence, The University of Tokyo, Bunkyo-ku, Tokyo 113-0033, Japan*

## I. EXECUTION TIME

TABLE I. Elapsed time for each method.

| Method | Elapsed Time [s] |
|---|---|
| Simulated Annealing | $1.62 \times 10^2$ |
| Quantum Annealing (Total) | $5.72 \times 10^2$ |
| QA (Computation Time) | 1.47 |

Here we discuss the execution times of the numerical experiments in this study. For a system of $L = 50, \alpha = 0.8$ and $\eta = 1$, we measure the time required to compute the single-step traffic signal states of $\sigma_i$ $(i = 1, \ldots, L^2)$. The quantum annealing approach involves communication time and job waiting time, that is, we have to send the locally generated problem to 2000Q physically located in Canada, via HTTP protocol and wait for 2000Q to return the problem. We record both the time including such communication and the time purely required for the quantum annealing to find the solution. The result is shown in Table I. Here, the listed values are the time required for one-step calculation, averaged over 200 samples. For the simulated annealing method, it takes $1.62 \times 10^2$ seconds to calculate one-step signal states. In contrast, the quantum annealing takes $5.72 \times 10^2$ seconds in total. The latter includes the communication time mentioned above, and the pure computation time is only 1.47 seconds. This is almost two orders of magnitude smaller than the simulated annealing method, which suggests the possibility of controlling traffic signals in real time.

* daisuke-inoue@mosk.tytlabs.co.jp
